# Requests for support by pregnant women with eating disorder symptoms: a systematic literature review of qualitative studies

**DOI:** 10.1186/s40337-025-01251-9

**Published:** 2025-04-24

**Authors:** Cecilia Brundin Pettersson, Klara Lundvik, Martina Isaksson, Mia Ramklint

**Affiliations:** 1 Department of Medical Sciences, Child and Adolescent Psychiatry, Uppsala University, Uppsala, Sweden; 2 Center for Clinical Research Dalarna, Uppsala University, Region Dalarna, Box 712, 79129 Falun, Sweden

**Keywords:** Eating disorders, Pregnancy, Qualitative studies, Support, Review

## Abstract

**Background:**

During the peripartum period, four to 13 percent of women may be affected by eating disorders (ED). Previous reviews of qualitative studies in pregnant women with ED have mainly focused on the women’s experiences during pregnancy and not on their expressed needs. This systematic review aimed to identify which types of support were requested by pregnant women with ED.

**Methods:**

The review was conducted in accordance with the “Enhancing transparency in reporting the synthesis of qualitative research” (ENTREQ) guidelines. Search for studies published between 1/1 2011- 14/3 2023 and 14/3 2023–9/1 2025, were performed in the following databases: PubMed, CINAHL, PsycInfo and Scopus. Studies were included if (1) the study population was pregnant women with ED symptoms /ED/ problems with food and eating, and (2) the study was an original qualitative study, and (3) the article was written in English. Rayyan, the AI-powered tool for systematic reviews, was used. Inclusion criteria were based on the SPICE-format. The CASP tool was used to assess quality in the included studies. Selected studies were read and critically appraised by two independent reviewers and a descriptive synthesis was conducted of expressed wishes for support based on quotes from the included studies. This review was preregistered in Prospero, 1/9 2023, (CRD42023456326).

**Results:**

Of 992 studies, only five fulfilled the inclusion criteria. From these studies three themes emerged: wish for support from health care, wish to get support from a partner and wish to use self-help strategies.

**Conclusion:**

This review found a knowledge gap regarding the type of support requested by pregnant women with ED symptoms.

**Plain English summary:**

In this study, we aimed to explore the existing literature on the needs of support expressed by pregnant women with eating disorders. We reviewed studies published between 2011 and 2025, and found only five that partially addressed these needs. Three main themes emerged: wish for support, self-help strategies, and support from a partner. Our findings showed that the women expressed a desire for their midwives to have enough knowledge about eating disorders to bring up the topic and talk about it with them. We also identified a gap in the literature, highlight the need for more qualitative research to better understand the specific support these women want and need during pregnancy. The role of the partner in providing support should also be addressed in future research.

**Supplementary Information:**

The online version contains supplementary material available at 10.1186/s40337-025-01251-9.

## Introduction

The lifetime prevalence of any eating disorder (ED) diagnosis in women has been estimated to be 8.4% (3.5–18.6%) [9]. Research suggests that ED symptoms continue during and after pregnancy, with approximately 4–7.5% of pregnant and 13% of postpartum women reporting impairing ED symptoms, where some women fulfil criteria for a specified ED [6, 24].

Eating disorders during pregnancy are associated with higher risks of a number of adverse pregnancy outcomes, both for the women and their offspring [15, 16]. These include both low and high birth weight in relation to gestational age, prematurity, miscarriage, increased risk for requiring a caesarean section, and various other obstetric complications [4, 15, 19]. Moreover, depression and anxiety are also more common in women with ED, both during pregnancy and postpartum [17]. Maternal nutritional status during pregnancy can have long-term effects on the energy balance in the offspring and is predictive of juvenile obesity, diabetes and increased risk for neurodevelopmental disorders [1, 16, 21, 33].

There are five previous reviews of qualitative studies exploring women’s experiences of EDs during the peripartum period [8, 27, 34–36]. Kaß focused on the impact of maternal ED on breastfeeding and Thompson on specific risk factors for ED symptoms during the postpartum period [13, 34]. Tierney and Fogarty examined women’s experiences of ED during pregnancy; Tierney [36] included studies published between January 1980 and October 2011 and Fogarty [8] included articles published up until 2015.

The pregnant women in the reviews by Tierney and Fogarty described their experiences of ED as internal conflicts, characterized by disturbing thoughts and emotions related to their body weight, alongside the simultaneous desire to be a good mother, which includes eating appropriately. These conflicts resulted in experiences of both fear and guilt, described as an “inner chaos” arising as a result of their strong ambivalence between wanting to eat for their foetus to grow and, at the same time, experiencing a need to control their body weight [8]. Another concern was about how others would judge their behaviour [36]. Fogarty described how the character of the eating disorder changed during pregnancy, although post-partum the majority of the women quickly returned to their pre-pregnancy eating disorder behaviours and thoughts [8]. Both of these qualitative reviews [8, 36] highlighted pregnancy as a particularly beneficial opportunity for women to come to terms with ED symptoms; however, neither Fogarty nor Tierney focused on studies that had reported women’s requests for support during this period, even if some needs were expressed. There have been calls for more studies to improve our understanding of experiences and needs in patients with ED [26], and the knowledge gap regarding care for women with EDs during the peripartum period has also been highlighted [2, 28, 29].

## Aim

The aim of this study was therefore to conduct a systematic review of qualitative studies to identify which support pregnant women with EDs request.

## Materials and methods

This review was conducted in accordance with guidelines for systematic reviews of qualitative research, namely “Enhancing transparency in reporting the synthesis of qualitative research” (ENTREQ) [37]. It was registered on 1 st September 2023 in the “International prospective register of systematic reviews” (PROSPERO) [25], (https://www.crd.york.ac.uk/prospero/display_record.php?RecordID=456326). The inclusion criteria were based on the SPICE-format. See Table 1.

**Table 1:** Research questions formulated according to the SPICE format

S = Setting	Maternal health care, primary care, specialized eating disorder care, specialist maternal care, self-help, support from relatives
P = Perspective	Pregnant women with subjective eating disorder symptoms or eating disorders
I = Interest	Women’s formulated wishes for support/treatment
C = Comparison	Not applicable
E = Evaluation	What type of support did they want? From whom did they want support? What type of treatment did they want?

### Search strategy and inclusion criteria

The search was based on a predetermined strategy and was performed on 14 th March 2023, with a complementary search performed on 9:th of January 2025. Since the purpose was to capture all relevant literature, a broad search was chosen with the emphasis on sensitivity. Inclusion criteria were: (1) the study population was pregnant women who had ED symptoms/ED/problems with food and eating during pregnancy. During the work it was decided to also include previously pregnant women with ED based on the assumption that their view of need for help during pregnancy, even if recalled, would still be relevant, (2) the study was an original qualitative study, and (3) the article was written in English.

The focus of the search was original articles published in scientific journals between 1 st January 2011 and 14 th March 2023, and the complimentary search from 14 th March 2023 to 9:th of January 2025. This timespan was chosen based on the previously performed systematic reviews, which included articles published between 1980 and 2015, in order to cover the literature published after these reviews [8, 36]. The complementary search was done during the process of revision to optimize for finding more relevant studies. To minimize the risk that articles were missed, it was decided to ensure that there was a proper overlap with previous reviews regarding the time frame for searches, which is why searches were started in 2011 instead of 2015.

The search string was based on the broad search strings from previous systematic reviews [8, 36] with the following search terms: “eating disorder” OR “anorexi” OR “bulimi” OR “disordered eating” OR “eating distress” OR “shape concern” OR “weight concern” OR “eating concern” OR “pregnant women” OR “mothers” OR “maternal health” OR “peripartum period” OR “maternal health services” (see Appendix 1, Table 1 for all searches). Searches were performed in four databases: PubMed, CINAHL, PsycInfo and Scopus.

### Selection of included studies

**Fig. 1:**
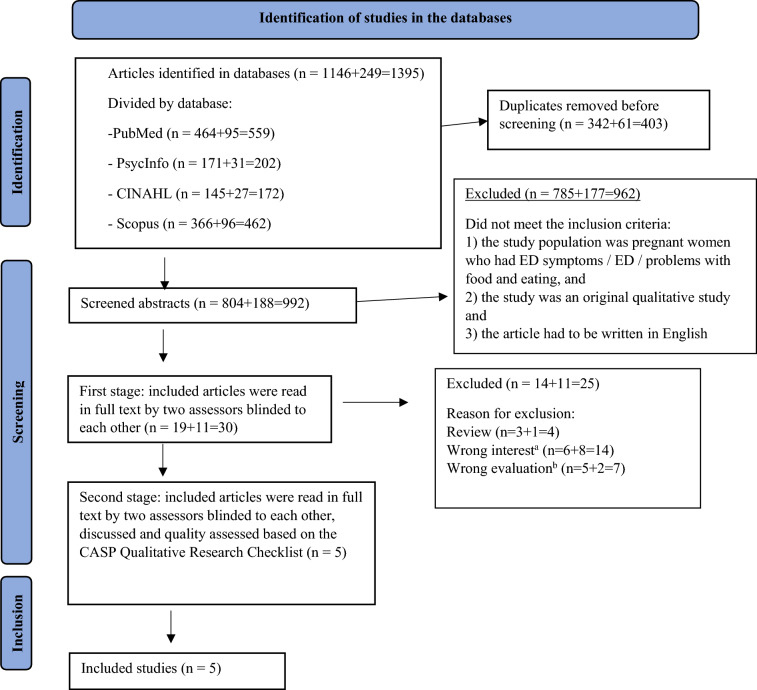
Flow chart of identification, screening, exclusion and inclusion of studies for this review. Databeses were searched at two occasions, with different time-periods, 1/1 2011–14/3 2023 and 14/3 2023–9/1 2025, results are separated in the flow-chart. Exclusion was based on the SPICE criteria, ^a^Interest: women’s formulated wishes for support/treatment, ^b^Evaluation: what type of support did they want? From whom? What type of treatment? ^c^Perspective: pregnant women with eating disorder or previously pregnant women with eating disorder

The searches identified 1395 studies, of which 403 duplicates were removed. The authors CBP and MR separately screened the abstracts of the remaining 992 studies using the AI-powered tool for systematic reviews Rayyan [23]. The same authors read all titles and abstracts independently and were blind to each other. The abstracts that were considered to meet the inclusion criteria by one or both assessors were then selected for further evaluation. There were few studies with currently pregnant women, and when studies were also included with women who had been pregnant with an ED but reported about it afterwards, there were still very few studies found. After the 30 articles included by at least one of the assessors had been retrieved and read in full-text, CBP and MR discussed them and five were included for further review and quality assessment. See Fig. 1 for PRISMA flow chart.

All five included articles are presented in Table 2.

**Table 2:** Included articles (n = 5) presented by title, aim, sample size and ED diagnoses, recruitment and assessment of EDs and method used for analysis

Author (year) title	Aim	Sample size and ED characteristics	Recruitment and assessment of ED	Method of analysis
Bye et al. [3] [3] Barriers to identifying eating disorders in pregnancy and in the postnatal period: A qualitative approach	To understand the barriers to disclosure and identification of ED in pregnancy and postnatally as perceived by women with a past or current ED	N = 101 n = 9 pregnant n = 36 ED during pregnancy AN = 34 BN = 16 BED = 24 ENDOS = 25	Recruitment: Women were recruited via a national parenting website; “Netmums” Assessment: Self-reported ED during or around the time of pregnancy, no specific instrument reported	Mixed-measures survey Thematic analysis
Tierney et al. [35] [35] Treading the tightrope between motherhood and an eating disorder: A qualitative study	To understand women’s experiences of pregnancy and motherhood whilst also having or having had an ED, exploring how one might impact on the other To understand these women’s perceptions of support whilst pregnant and in the early months of a child’s life To understand these women’s experiences of caring for a new infant	N = 8 n = 3 pregnant BN = 1 AN = 5 Restrictive eating = 2	Recruitment: Women were recruited via midwives in maternal health care and via an ED organisation's website Assessment: Self-reported ED symptoms: SCOFF (Morgan et al., 1999) Eating disorder Screen for Primary care (ESP)(Cotton et al., 2003)	Framework analysis of transcripts
Claydon et al. [5] [5] Waking up every day in a body that is not yours: a qualitative research inquiry into the intersection between eating disorders and pregnancy	Describe the concerns that women with ED feel when becoming pregnant Understand some of the unique barriers in prenatal care that women with ED face Learn how women with ED can be better supported throughout pregnancy to improve both maternal and child health outcomes	N = 15 n = 9 pregnant AN = 4 BN = 3 EDNOS/OSFED = 2	Recruitment: Personal referral and Facebook Assessment: Self-reported ED, diagnoses, based on evaluation at a clinic, but no specific instrument reported	Thematic analysis
Mason et al. (2012) [18] The experience of pregnancy in women with a history of anorexia nervosa: An Interpretive Phenomenological Analysis	Explore the experience of pregnancy for women who have a history AN, in relation to the impact of AN on pregnancy and of pregnancy on AN	N = 6 n = 4 pregnant AN = 5 Previous AN = 1	Recruitment: via regional and national ED specific websites, newsletters and groups, and via general advertising Assessment: The Structured Clinical Interview for DSM Disorders, Patient Version (SCID; First, Spitzer, Gibbon & Williams, 2002)	Interpretative phenomenology analysis (IPA)
Stitt & Reupert [30] [30] Mothers with an eating disorder: “food comes before anything”	Identify parents’ perceptions regarding the impact of the ED on their children and parenting	N = 9 all postnatal AN = 4 BN = 4 EDNOS = 1	Recruitment: repeated advertisements posted on support group websites for those with an ED and in local state newspapers Assessment: Self-reported ED diagnoses, based on evaluation at a clinic, but no specific instruments reported	Interpretative Phenomenological Analysis (IPA)

### Quality assessment

A checklist for the assessment of qualitative studies, the Critical Appraisal Skills Programme (CASP), was used [20] to evaluate the risk of bias in the five articles analysed. Two of the authors, CBP and MR, independently evaluated the CASP criteria for the five included articles [3, 35]. Disagreements were discussed until consensus was reached. Four included articles were considered to be of high quality, one article was considered to be of moderate quality, see Appendix Table 2.

### Synthesis method

All themes with all provided quotes from the included articles were extracted. They could be relevant for the study question or not, but were still extracted, see Appendix Table 3. Many of the quotes were about obstacles for help-seeking or descriptions of perceived vulnerability that can be relevant for care providers intending to provide support but were not about requested support, and other quotes expressed no wish for support or clearly stated that they didn´t want any help or focused on other problems. None of these quotes were included in our synthesis, see Table 3 for the relevant quotes included in the synthesis. The quotes concerning type of support that pregnant women with ED symptoms did request were discussed by all authors and grouped into themes, see Table 3. The analysis was performed as a descriptive synthesis providing a compilation of the results with a minimal degree of interpretation.

**Table 3:** Themes and quotations extracted from included articles in a review of qualitative studies about wanted support expressed by pregnant women with eating disorders (ED). Quotes from the included articles were recoded into themes concerning wanted support during pregnancy with an ED. The themes were; ‘Wish för support from health care’, ‘Wish for Support from a partner’ and ‘Wish to use Self-help strategies’

Article	Quotations from included articles expressing wishes for support	Themes concerning wanted support
Bye et al. [3] [3] Barriers to identifying eating disorders in pregnancy and in the postnatal period: A qualitative approach	*“I didn’t have the same midwife for long enough to speak to them, it was rather stressful and upsetting”*	Wish for support
	*“I don’t like to talk about it and think I can manage on my own”*	Self-help strategies
	*“I just wanted to deal with it myself”*	Self-help strategies
Tierney et al. [35](2011) Treading the tightrope between motherhood and an eating disorder: A qualitative study	*‘‘I really, really wanted to be able to eat more in order to produce better milk and I couldn’t do it and I felt that I’d failed him. I can remember crying when I was buying the formula for the first time and I couldn’t...be around when he was given the formula because it upset me...I didn’t want anyone else to know he was getting the formula. There was only me and my husband that knew we were giving it to him.’’*	Whish for support
	*‘‘I’d stopped like proper exercising, weights and biking and stuff like that at about six months and then I thought OK we’ll just see this as a little retirement. You can start it all up again once the baby’s here. And I just let go fullstop then. I just thought OK just monitor your weight, make sure you don’t put too much on and just be relaxed about it all. You can work at making it all better once [daughter] turns up.’’*	Self-help strategies
Claydon et al. [5] [5] Waking up every day in a body that is not yours: a qualitative research inquiry into the intersection between Eating Disorders and pregnancy	*“I certainly felt a lack of … communication between psychiatric care and maternity care and needing some sort of, it doesn't have to be a specialized midwife but just someone who can cross barriers and help you navigate your way through the pregnancy from both perspectives and not just one or the other.”*	Wish for support
	*“Fortunately, my husband, when they were in second grade and third grade … because I was making like bizarre food. He completely took over food. He brought in chips. He normalized food for my girls. I think that probably saved them, considering that my own biological mother had an eating disorder and then I did.”*	Support from a partner
	*“somebody could know and do the checks, but it’s information that I’d rather not know.”*	Wish for support
	*“I completely threw myself relentlessly into school at that point … because I had to distract myself somehow.”*	Self-help strategies
	*“I forced myself to not keep track of things anymore … because at the end of day I knew I had to be consuming at least 2300 cal and if I hit that, I’d be upset, but then if I didn’t hit that, I’d be upset. So, either way, you’re never going to win in that situation … I just had to give up accountability all together and just say fuck it.”*	Self-help strategies
	*“I don’t trust myself to make the best decisions regarding pregnancy. Maybe I would and I’m underestimating myself, but I would rather just have the outside support.”*	Wish for support
	*“I go to the bathroom at the end of the meal and it's like I have to remind myself not to do it. I choose not to because I know it's not healthy, but it's a choice that I make.”*	Self-help strategies
Mason et al (2012) [18] The experience of pregnancy in women with a history of anorexia nervosa: An Interpretive Phenomenological Analysis	*“Um… the only thing that I did manage to do, mostly, was give up taking laxatives.”*	Self-help strategies
	*“Yeah, I did struggle with the changing body, yeah, I did find that hard…, but at the end of the day I knew that was what I wanted. To have kids. So I had to motivate myself to do it.”*	Self-help strategies
	*“…You’re not really thinking about anyone else, and all you think about is food, and so… um… I just had to stop being like that, it was like ‘‘well I can’t think about myself anymore, I’ve got a baby that I need to, that needs to develop and it needs to be born and it needs to be perfect, and so I can’t think about myself anymore.”*	Self-help strategies
	*“And it wasn’t just my baby, it was husband’s baby as well, and I didn’t want to let anyone down…”*	Self-help strategies
	*“I didn’t react to [the feelings], that’s the important thing. I didn’t let them fester too much. I think the feelings were there, but I didn’t respond to them…”*	Self-help strategies
	*“And the sort of eating thing, and how I eat, that could just sort of be kept on the backburner until after I’d had the baby. And then I’d start thinking about it again and how I was going to get, you know, lose the weight and get back to how I was before.”*	Self-help strategies
	*“I think I almost… every time a feeling crept in, I almost saw it as a bag that I put to one side for the time being. It was like, you know, that’s an issue for later, that’s going to have to be dealt with later…”*	Self-help strategies
	*“I was fighting the ED more, I was more prepared to challenge it, and I was more worried about the consequences”*	Self-help strategies
	*“I suppose it I do it now as a way of, well, just punishing myself and things like that, but I suppose when you’re pregnant you don’t want to punish the child as well.”*	Self-help strategies
	*“I was OK with the changes which I could directly attribute to being pregnant. I was fine with having a big bump, and I quite liked that. Um… but I wasn’t OK with putting weight on anywhere else at all… …I was happy to look pregnant, and I wanted to look pregnant, I just didn’t want to have any fat anywhere else, on my legs, or arms, or face, anything like that…”*	Self-help strategies
	*“I just knew that that was going to be the precious, the most precious thing to look after, and actually my body was sup- porting and nurturing a little person in there, and that was more important to me than anything.”*	Self-help strategies
	*“So I didn’t get any help. And I did actually say to my doctor as well, I remember saying to him I felt really depressed and low, and I wasn’t offered any help… I felt as if, ‘do people really believe me here? Do people believe that I feel…?’… And so I felt, yeah, I felt wretched… Yeah I didn’t feel good. I felt completely and totally miserable when I was pregnant.”*	Wish for support
	*“I guess I felt as if… I had been abandoned to be honest… I wasn’t asking for a lot, I think. And that’s the thing that I feel so let down about. I wasn’t asking for a lot, I was just asking for a phone call, or someone to just pop by and see me, and just sit and chat for an hour, you know. I just wanted some contact with the outside world, and I’m not exaggerating when I say that just didn’t happen at all.”*	Wish for support
	*“…a lot of what the midwives give you is geared towards staying active and not gaining too much weight during pregnancy, and all the health problems which could be caused by gaining too much weight. It’s aimed at the general population, and I can see that now. But… I think when you’re in the middle of an eating disorder, you could sort of use it to think ‘‘well, it’s just as unhealthy if I gain all this weight, and if I gain weight I’ll have gestational diabetes and pre-eclampsia and all these things”.”*	Wish for support
Stitt & Reupert (2014) [30] Mothers with an eating disorder: “food comes before anything”	*“…. directed at younger people and not older people... yeah there isn’t [aren’t] methods that include family life”*	Wish for support
	*“meal plans within the context of family life” “I can’t set my meal plans …. when you’ve got kids your life isn’t your own to manage as you would like ….”*	Wish for support
	*“It’s not appropriate for me as an adult to have my mother come and take control of my food, and while my husband, I guess, was willing and able to do that, that’s sort of not really an appropriate balance in the relationship either.”*	Support from a partner
	*“…. therapists don’t quite get that everybody doesn’t have babysitters they can ring up at short notice …. I actually had one woman who …. basically said ‘If you can’t commit to come every single week then I can’t treat you’ and I said ‘Look, I’m trying but if my kids are sick, what do I do’ ….”*	Wish for support

## Results

All included articles are presented in Table 2, together with information about how many of the women that were currently pregnant, their reported EDs, how they were recruited and if assessment of EDs were described. Bye et al. [3] tried to understand the barriers to disclosure of ED as perceived by pregnant women. In their study, 101 pregnant and postnatal women (9% pregnant) with current or past ED completed a mixed-measures survey. Tierney et al. [35] aimed to understand the experiences of pregnancy in women with ED, their perceptions of support, and their experiences of caring for their infant. They interviewed eight women, three of whom were pregnant. Claydon et al. [5] interviewed 15 women, including both those with past pregnancies and some who had never been pregnant but had an ED. The aim was to explore the intersection of ED and pregnancy from perspectives of women with a history of ED. In the study of Mason et al. [18] six women with a history of anorexia nervosa (AN) were interviewed to explore their experiences of pregnancy. Stitt et al. [30] interviewed nine mothers with various EDs, with the aim to identify parents’ perceptions regarding the impact of the ED on their children and parenting. The themes and quotes presented in the included articles related to requested support were very few and mainly focusing on self-help strategies, see Appendix Table 3. Among the quotes it was possible to identify three implicit themes on this subject: *Wish for support from health care, Wish to get support from a partner and Wish to use self-help strategies,* all three themes are presented in Table 3.

In all studies women expressed at least some wish for support from the health care system [3, 5, 18, 30, 35], and in two studies [5, 30] there were expressed wishes for support from a partner, even if one of them was in relation to feeding older children. Strategies to handle the ED in a constructive way but by themselves, not asking for any help, was most commonly expressed, here interpreted as a wish to do so. There were several quotes interpreted as not wanting any help, or descriptions of obstacles for help-seeking or descriptions of perceived vulnerability. Since they didn´t answer the study question, they were not further analysed, but provided in Appendix Table 3.

### Wish for support

Some women experienced a lack of opportunity to tell their midwives about their ED [3, 35], or tried to reach out to their doctor but wasn´t understood [18], maybe because they talked about feeling bad and not being specific about their ED. This wish to tell their midwife was interpreted as a step towards reaching out for help.

*“I didn’t have the same midwife for long enough to speak to them, it was rather stressful and upsetting”*[3]

*“So I didn’t get any help. And I did actually say to my doctor as well, I remember saying to him I felt really depressed and low, and I wasn’t offered any help… I felt as if, ‘do people really believe me here? Do people believe that I feel…?’… And so I felt, yeah, I felt wretched… Yeah I didn’t feel good. I felt completely and totally miserable when I was pregnant.”* [18]

Women asked for being checked without being informed about the results [5]. They experienced getting inappropriate information from their midwifes, information better suited for pregnant women without an ED [18]. Finally, they suggested a better communication between psychiatric care and maternity care, and suggested that this communication should be provided by a professional appointed for being the link between them, such as a specialised midwife [5].

*“somebody could know and do the checks, but it’s information that I’d rather not know.”* [5]

*“…a lot of what the midwives give you is geared towards staying active and not gaining too much weight during pregnancy, and all the health problems which could be caused by gaining too much weight. It’s aimed at the general population, and I can see that now. But… I think when you’re in the middle of an eating disorder, you could sort of use it to think ‘‘well, it’s just as unhealthy if I gain all this weight, and if I gain weight I’ll have gestational diabetes and pre-eclampsia and all these things”* [18]*.*

*“I certainly felt a lack of … communication between psychiatric care and maternity care and needing some sort of, it doesn't have to be a specialized midwife but just someone who can cross barriers and help you navigate your way through the pregnancy from both perspectives and not just one or the other.”*[5]

### Support from partner

Some women described wanting practical support from their partner, e.g. cooking meals and doing the grocery shopping [35], even if this was also problematized because of the potential negative impact this could have on their relationship [30].

*“It’s not appropriate for me as an adult to have my mother come and take control of my food, and while my husband, I guess, was willing and able to do that, that’s sort of not really an appropriate balance in the relationship either.”*[30]

*“Fortunately, my husband, when they were in second grade and third grade … because I was making like bizarre food. He completely took over food. He brought in chips. He normalized food for my girls. I think that probably saved them, considering that my own biological mother had an eating disorder and then I did.”*[5]

### Self-help strategies

Many quotes were categorized as wish to use self-help strategies, see Table 3. Sometimes the quotes could have been interpreted as no wishes for help, but when the intention was interpreted as a way of managing the ED in a constructive way, they were classified as self-help strategies, see Table 3. Women described how they wanted to do the right things for their child by using self-help strategies, such as distraction [35]. Other strategies included changing their relationship to eating, such as eating an extra meal per day and eating new types of food [35]. They described cognitive strategies, how they motivated themselves by taking the child´s perspective or the partner´s perspective [18].

*“I forced myself to not keep track of things anymore … because at the end of day I knew I had to be consuming at least 2300 cal and if I hit that, I’d be upset, but then if I didn’t hit that, I’d be upset. So, either way, you’re never going to win in that situation … I just had to give up accountability all together and just say fuck it.”* [5]

*“Yeah, I did struggle with the changing body, yeah, I did find that hard…, but at the end of the day I knew that was what I wanted. To have kids. So I had to motivate myself to do it.”*[18]

*“And it wasn’t just my baby, it was husband’s baby as well, and I didn’t want to let anyone down…”* [18]

## Discussion and conclusions

The main finding was the lack of studies exploring requested support during pregnancy by women with EDs. While previous reviews have focused on the experiences of women with an ED of being pregnant, the current systematic literature review is focusing on what types of support pregnant women with EDs ask for. There were only five articles that fulfilled the inclusion criteria, although no article included only pregnant women [3, 5, 18, 30, 35]. Most studies interviewed women with experience of ED during pregnancy but not being currently pregnant. Whether the women’s perspectives change between pregnancy and postpartum is not known, but it is clear that there is a lack of studies that ask pregnant women with EDs about their needs. The searches were very broad with the intention to capture all articles published, and even if EDs were operationalised as ‘ED symptoms, EDs, or problems with food and eating, and even if previously pregnant women were included, there were still very few articles found.

The previous review by Fogarty et al. [8] reported that pregnant women want to be asked directly about their ED symptoms, for example wanting their midwives to ask about them, and show an interest in and have knowledge about ED, supported by this review. Moreover, Fogarty et al. also reported that some women described a lack of appropriate care for their ED, even in cases where they did disclose their symptoms [8]. Similarly, women that already had children described how the care that was offered had not been adapted to women of their age with a family life and children [30]. Moreover, in the article by Mason et al. [18], included in this review, it was described, but without quotes, a wish for contact with a dietitian at a specialist ED service who schematically showed how much weight the woman would gain through the different phases of pregnancy.

In accordance with this review, Fogarty et al. [8] found that the women themselves did not express needs concerning the type of support they wanted during pregnancy [8]. [36]The importance of seeing pregnancy as a time of change where women are more open to addressing their problems has been highlighted [32]. Fogarty et al. [8] also point out that pregnancy is a time when women can feel a loss of control and increased anxiety, which might explain the internal “tug-of-war” that many women express experiencing, vacillating between doing the right thing for the child but also meeting the needs of the ED. Some women in the articles by Claydon et al. and Bye al. [3, 5] experienced that there was a lack of communication between psychiatric and maternal care regarding helping them to navigate their way through pregnancy. Some reported not seeing the same midwife for long enough to be able to gain the trust that they needed to tell them about their problems.

Identifying self-help strategies used by pregnant women with ED symptoms and supporting them in using these strategies could be empowering, and a potentially effective intervention. The use of self-help strategies was described in the studies reviewed.

Both emotional and practical support from their partner were described by women as being wanted. This was reported in a previous review [36], which also discussed the partner’s role in supporting the pregnant woman and what support the partners themselves need to be able to support their pregnant partner. According to the review by Tierney et al. [36], there is a lack of knowledge about how to support the partner of a pregnant woman with an ED. The partner is often torn between the best interest of the foetus and a wish to support the woman.

Postpartum women expressed a need for peer support, for example from a group of other women in the same situation [18]. They reported experiencing isolation or withdrawing from others as a result of their ED [18, 30, 35]. Studies of non-pregnant patients have shown that group treatment for EDs, mainly bulimia nervosa (BN) and binge eating disorders (BED), have an effect [11]. This should be evaluated in pregnant women, and include an exploration of their perceptions of participating. The power of peer support in various health conditions, for both patient and family members, has been highlighted [22]. Peer support in group settings for families with children with ED has been experienced as helpful and meeting persons with similar experiences has also been described as valuable [10]. Peer support for both the pregnant woman with ED symptoms, as well as her partner and family, could supplement the care of these women.

The majority of articles about ED during pregnancy conclude that these pregnancies should be considered high-risk [7, 14, 15]. Even if the experiences of pregnancy in women with ED symptoms have been explored [3, 5, 35], their wishes for support have not been explored to the same extent.. The importance of paying these women more attention is emphasized in the literature, as is exploring the support they wish for [8, 29, 31]. This review found there was a lack of studies, and therefore a knowledge gap, regarding what type of support is wanted by pregnant women with ED symptoms. Patient-centred care should consider the needs of patients from their own perspectives; more patient-centred research within this field is therefore required.

## Strengths and limitations

Limitations of this study include the narrow focus on expressed needs by pregnant women which resulted in only a few included studies. The number of papers found is not sufficient to provide conceptual insight and more research is needed. However, this can also be a strength since it revealed the large knowledge gap within the literature regarding the type of support that these women ask for. Inclusion criteria for what EDs that could be included was very broad; having ED symptoms, EDs, problems with food and eating during pregnancy, and it was not mandatory that the EDs were assessed with diagnostic interviews, in fact it was only one of the included studies that used one. However, suffering from eating disorder symptoms is not delimited by the categorical ED diagnoses, since almost half of help-seeking patients doesn´t fulfil criteria for any specific ED [12, 38].

## Clinical implications

Staff in maternal healthcare need to ask the women questions about ED symptoms during routine antenatal care, thereby allowing the women to articulate their need for support.

## Areas for future research

There is a need for more qualitative research about the support that pregnant women with ED request. It is important to investigate what role the woman’s partner can play in providing support and what needs the partners express to be able to provide support. It is also important to know if the women’s perspectives change during the peripartum period.

## Supplementary Information

Additional file 1

## Data Availability

No datasets were generated or analysed during the current study.
