# Supplementary material for: Requests for support by pregnant women with eating disorder symptoms: a systematic literature review of qualitative studies
Source: J Eat Disord. 2025 Apr 24;13:71. doi: 10.1186/s40337-025-01251-9 (PMC12023643; doi:10.1186/s40337-025-01251-9)
Supplement: Supplementary file 1 — Additional file 1 [file 40337_2025_1251_MOESM1_ESM.docx]

**Appendix**

**Table 1 Search string for use in PubMed, CINAHL, APA PsykInfo and Scopus 14-03-2023 and 09-01-2025**

**Database:** PubMed. Date: 14-03-2023 and 09-01-2025

|  | Terms | Results  14-03-2023 | Results  09-01-2025 |
| --- | --- | --- | --- |
| Concept 1, #1 | "Anorexia"[mh] OR "Bulimia"[mh] OR "Feeding and Eating Disorders"[mh] | 43,174 | 46,039 |
| #2 | "eating disorder*"[tiab] OR "anorexi*"[tiab] OR "bulimi*"[tiab] OR "disordered eating"[tiab] OR "eating distress"[tiab] OR "shape concern"[tiab] OR "weight concern"[tiab] OR "eating concern"[tiab] OR "EDNOS"[tiab] OR "UFED"[tiab] OR "OSFED"[tiab] OR (("bing*"[tiab] OR "restraint*"[tiab]) AND ("feed*"[tiab] OR "food*"[tiab] OR "eating"[tiab] OR "diet*"[tiab])) | 65,152 | 71,876 |
| #3 | #1 OR #2 | 75,483 | 82,485 |
| Concept 2, #4 | "Pregnancy"[mh] OR "Pregnant Women"[mh] OR "Mothers"[mh] OR "Maternal Health"[mh] OR "Peripartum Period"[mh] OR "Postpartum Period"[mh] OR "Maternal Health Services"[mh] | 1,065,496 | 1,125,005 |
| #5 | "pregnan*"[tiab] OR "perinatal"[tiab] OR "peri natal"[tiab] OR "peripartum"[tiab] OR "peri partum"[tiab] OR "prepartum"[tiab] OR "pre partum"[tiab] OR "prenatal"[tiab] OR "pre natal"[tiab] OR "antenatal"[tiab] OR "ante natal"[tiab] OR "antepartum"[tiab] OR "ante partum"[tiab] OR "postpartum"[tiab] OR "post partum"[tiab] OR "postnatal"[tiab] OR "post natal"[tiab] OR "mother*"[tiab] OR "maternal"[tiab] | 1,088,340 | 1,181,900 |
| #6 | #4 OR #5 | 1,488,353 | 1,488,353 |
| Concept 3, #7 | "Empirical Research"[mh] OR "Interview"[pt] OR "Interviews as Topic"[mh] OR "Personal Narrative"[pt] OR "Personal Narratives as Topic"[mh] OR "Focus Groups"[mh] OR "Narration"[mh] OR interview*[tiab] "qualitative"[all fields] OR questionnaire*[all fields] OR "ethnological research"[all fields] OR ethnograph*[all fields] OR ethnonursing[all fields] OR phenomenol*[all fields] OR "grounded theor*"[all fields] OR "grounded stud*"[all fields] OR "grounded research"[all fields] OR "grounded analys*"[all fields] OR "life stor*"[all fields] OR "women's stor*"[all fields] OR "focus group*"[all fields] OR account[tiab] OR accounts[tiab] OR narrative*[tiab] OR narration[tiab] OR "conversation analys*"[tiab] OR "personal experience*"[tiab] OR "lived experience*"[tiab] OR "life experience*"[tiab] OR "discourse analys*"[tiab] OR "discursive analys*"[tiab] OR "narrative analys*"[tiab] | 1,671,828 | 1,898,751 |
|  | #3 AND #6 AND #7 | 782 | 875 |
|  | #3 AND #6 AND #7  2011-2023 | 464 | 95 |

#7 Modified filter from: <https://searchfilters.cadth.ca/list?q=&p=1&ps=20&topic_facet=qualitative%20studies%20000000%7CQualitative%20studies>

**Database:** CINAHL via EBSCO. Expanders: Apply equivalent subjects, Search modes: Find all my search terms. Date: 14-03-2023 and 09-01-2025

|  | Terms | Results  14-03-2023 | Results  09-01-2025 |
| --- | --- | --- | --- |
| Concept 1, S1 | (MH "Eating Disorders+") | 20,948 |  |
| S2 | TI ( "eating disorder*" OR anorexi* OR bulimi* OR "disordered eating" OR "eating distress" OR ((shape OR weight OR eating) W0 concern) OR EDNOS OR UFED OR OSFED OR ((bing* OR restraint*) AND (feed* OR food* OR eating OR diet*)) ) OR AB ( "eating disorder*" OR anorexi* OR bulimi* OR "disordered eating" OR "eating distress" OR ((shape OR weight OR eating) W0 concern) OR EDNOS OR UFED OR OSFED OR ((bing* OR restraint*) AND (feed* OR food* OR eating OR diet*)) ) | 24,790 |  |
| S3 | S1 OR S2 | 29,950 |  |
| Concept 2, S4 | (MH "Pregnancy+") OR (MH "Mothers+") OR (MH "Maternal Health Services+") OR (MH "Maternal-Child Health") | 289,856 |  |
| S5 | TI ( pregnan* OR perinatal OR "peri natal" OR peripartum OR "peri partum" OR prepartum OR "pre partum” OR prenatal OR "pre natal" OR antenatal OR "ante natal" OR antepartum OR "ante partum" OR postpartum OR "post partum" OR postnatal OR "post natal" OR mother* OR maternal ) OR AB ( pregnan* OR perinatal OR "peri natal" OR peripartum OR "peri partum" OR prepartum OR "pre partum” OR prenatal OR "pre natal" OR antenatal OR "ante natal" OR antepartum OR "ante partum" OR postpartum OR "post partum" OR postnatal OR "post natal" OR mother* OR maternal ) | 317,372 |  |
| S6 | S4 OR S5 | 415,465 |  |
| Concept 3,  S7 | MH Qualitative Studies OR MH Grounded theory OR MH Narratives OR MH Interviews+ OR MH Audiorecording OR MH Focus Groups OR MH Discourse Analysis OR MH Ethnographic Research OR MH Ethnonursing Research OR MH Phenomenology OR MH Phenomenological Research OR MH Life Experiences+ OR TI qualitative OR AB qualitative OR TI interview* OR AB interview* OR TI ("ethnological research") OR AB ("ethnological research") OR TI ethnonursing OR AB ethnonursing OR TI ethnograph* OR AB ethnograph* OR TI phenomenol* OR AB phenomenol* OR TI "focus group*" OR AB "focus group*" OR TI (grounded N1 (theor* OR analys?s OR research OR studies OR study)) OR AB (grounded N1 (theor* OR analys?s OR research OR studies OR study)) OR TI ("life stor*") OR AB ("life stor*") OR TI ("conversation analys?s" OR "personal experience*") OR AB ("conversation analys?s" OR "personal experience*") OR TI ((life OR lived) N1 experience*) OR AB ((life OR lived) N1 experience*) OR TI ((discurs* OR discourse*) N3 analys?s) OR AB ((discurs* OR discourse*) N3 analys?s) OR TI ("narrative analys?s") OR AB ("narrative analys?s") | 543,650 |  |
| S8 | S3 AND S6 AND S7 | 289 | 313 |
| S9 | S8 Published Date: 20110101-20231231 | 145 | 27 |

S7 Modified filter from: <https://searchfilters.cadth.ca/list?q=&p=1&ps=20&topic_facet=qualitative%20studies%20000000%7CQualitative%20studies>

**Database:** APA PsycInfo via OVID <1806 to February Week 4 2023> and APA PsycInfo via OVID <1806 to December 2024 Week 5>

|  | Terms | Results  14-03-2023 | Results  09-01-2025 |
| --- | --- | --- | --- |
| Concept 1,  #1 | exp eating disorders/ | 34,682 | 38,635 |
| #2 | (eating disorder* or anorexi* or bulimi* or disordered eating or eating distress or ((shape or weight or eating) adj concern) or EDNOS or UFED or OSFED or ((bing* or restraint*) and (feed* or food* or eating or diet*))).ti,ab,id. | 46,930 | 51,220 |
| #3 | 1 or 2 | 49,179 | 53,707 |
| Concept 2,  #4 | exp pregnancy/ | 47,718 | 58,629 |
| #5 | exp mothers/ | 45,692 | 49,852 |
| #6 | expectant mothers/ | 964 | 1,172 |
| #7 | perinatal period/ or antepartum period/ or postnatal period/ | 10,159 | 12,585 |
| #8 | (pregnan* or perinatal or peri natal or peripartum or peri partum or prepartum or pre partum or prenatal or pre natal or antenatal or ante natal or antepartum or ante partum or postpartum or post partum or postnatal or post natal or mother* or maternal).ti,ab,id. | 226,030 | 242,010 |
| #9 | 4 or 5 or 6 or 7 or 8 | 237,018 | 253,769 |
| #10 | 3 and 9 | 2,690 | 2,873 |
| Concept 3,  #11 | Empirical Methods/ or exp Qualitative Methods/ or exp Interviews/ or Interviewing/ or Narratives/ or Storytelling/ | 70,796 | 77,960 |
| #12 | interview*.ti,ab,id. | 381,429 | 427,082 |
| #13 | qualitative.ti,ab,jx,id. | 214,561 | 253,682 |
| #14 | ethnological research.ti,ab,id. | 13 | 14 |
| #15 | ethnograph*.ti,ab,id. | 32,373 | 35,286 |
| #16 | ethnomedicine.ti,ab,id. | 60 | 62 |
| #17 | ethnonursing.ti,ab,id. | 72 | 75 |
| #18 | phenomenol*.ti,ab,id. | 51,807 | 59,335 |
| #19 | (grounded adj (theor* or study or studies or research or analys?s)).ti,ab,id. | 19,543 | 21,777 |
| #20 | life stor*.ti,ab,id. | 4,154 | 4,542 |
| #21 | (focus adj group*).ti,ab,id. | 43,880 | 50,803 |
| #22 | (conversation analys?s or personal experience*).ti,ab,id. | 16,143 | 17,712 |
| #23 | ((lived or life) adj experience*).ti,ab,id. | 35,196 | 42,676 |
| #24 | ((discourse* or discurs*) adj3 analys?s).ti,ab,id. | 10,652 | 11,805 |
| #25 | or/11-24 | 605,938 | 680,043 |
| #26 | 10 and 25 | 397 | 438 |
| #27 | limit 26 to yr="2011 -Current" | 171 | 31 |

#11-25 Modified filter from: <https://searchfilters.cadth.ca/list?q=&p=1&ps=20&topic_facet=qualitative%20studies%20000000%7CQualitative%20studies>

**Data base:** Scopus via Elsevier. Date: 14-03-2023 and 09-01-2025

|  | Terms | Results  14-03-2023 | Results  09-01-2025 |
| --- | --- | --- | --- |
| Concept 1, #1 | TITLE-ABS-KEY ( "eating disorder" OR "eating disorders" OR anorexi* OR bulimi* OR "disordered eating" OR "eating distress" OR "shape concern" OR "weight concern" OR "eating concern" OR ednos OR ufed OR osfed OR ( ( bing* OR restraint* ) AND ( feed* OR food* OR eating OR diet* ) ) ) | 157,409 | 173,926 |
| Concept 2, #2 | TITLE-ABS-KEY ( pregnan* OR perinatal OR "peri natal" OR peripartum OR "peri partum" OR prepartum OR "pre partum" OR prenatal OR "pre natal" OR antenatal OR "ante natal" OR antepartum OR "ante partum" OR postpartum OR "post partum" OR postnatal OR "post natal" OR mother* OR maternal ) | 1,900,747 | 2,067,184 |
| Concept 3, #3 | TITLE-ABS-KEY ( "Empirical Research" OR interview OR "Interviews as Topic" OR "Personal Narratives" OR "Focus Groups" OR narration OR interview* OR qualitative OR "ethnological research" OR ethnograph* OR ethnomedicine OR ethnonursing OR phenomenol* OR "grounded theor*" OR "grounded study" OR "grounded studies" OR "grounded research" OR "grounded analysis" OR "grounded analyses" OR "life stor*" OR ( focus W/3 group* ) OR "conversation analysis" OR "personal experience*" OR "conversation analyses" OR "lived experience" OR "life experience*" OR ( discourse* W/3 analysis ) OR ( discourse* W/3 analyses ) OR ( discurs* W/3 analysis ) OR ( discurs* W/3 analyses ) ) | 2,136,383 | 2,530,863 |
|  | #1 AND #2 AND #3 | 649 | 740 |
|  | #1 AND #2 AND #3  2011-2023 | 366 | 96 |

#3 Modified filter from: <https://searchfilters.cadth.ca/list?q=&p=1&ps=20&topic_facet=qualitative%20studies%20000000%7CQualitative%20studies>

**Appendix**

**Table 2** Quality assessment based on the CASP Qualitative Research Checklist (1-10*)

| Authors | 1 | 2 | 3 | 4 | 5 | 6 | 7 | 8 | 9 | 10 | Assessment |
| --- | --- | --- | --- | --- | --- | --- | --- | --- | --- | --- | --- |
| (Tierney et al. 2011) | Y | Y | Y | Y | Y | Y | Y | Y | Y | Y | High |
| (Bye et al. 2018) | Y | Y | Y | Y | Y | Y | Y | Y | Y | Y | High |
| (Claydon et al. 2018) | Y | Y | Y | Y | U | U | Y | Y | Y | Y | Moderate |
| (Stitt & Reupert 2014) | Y | Y | Y | Y | Y | Y | Y | Y | Y | Y | High |
| (Mason et al. 2012) | Y | Y | Y | Y | Y | Y | Y | Y | Y | Y | High |
|  |  |  |  |  |  |  |  |  |  |  |  |

* CASP criteria for qualitative studies (checklist): 1. Was there a clear statement of the aims of the research? 2. Was a qualitative methodology appropriate? 3.Was the research design appropriate to address the aims of the research? 4. Was the recruitment strategy appropriate to the aims of the research? 5. Were the data collected in a way that addressed the research issue? 6. Has the relationship between researcher and participants been adequately considered? 7. Have ethical issues been considered? 8. Was the data analysis sufficiently rigorous? 9. Is there a clear statement of the findings? 10. How valuable is the research? (Y=Yes, N=No, U=Unclear).

**Appendix**

**Table 3.** Themes and quotations extracted from included articles in a review o about wanted support expressed by pregnant women with eating disorder symptoms (ED). Quotes from the included articles were recoded into themes concerning wanted support during pregnancy with an ED. The resulting themes were; **Wish för support** from health care, Wish to use **Self-help strategies** and Wish for **Support from a partner.** Some quotes were coded **No wish for support**. The other categories were not answering our research question.

| **Author (year)**  **Title** | **Themes and quotations from included articles** | **Analysis** | |
| --- | --- | --- | --- |
|  |  | **Themes concerning wanted support** | **Other categories^a^** |
| Bye et al. (2018)    Barriers to identifying eating disorders in pregnancy and in the postnatal period: A qualitative approach | Five themes:   1. stigma   *“I was overweight according to my BMI. I didn’t think they would believe me to tell them I had an actual problem. I was patronised by more than one healthcare professional who tried to educate me on nutrition. I got the impression they thought I was just lazy and ate junk food all of the time when this wasn’t the case. I felt they were too judgemental to approach”*  *“I would have been to worried to discuss with my midwife etc. for fear of being reprimanded for it (i.e. referred to social services)”*   1. lack of opportunity   *“they didn’t ask and it wasn’t raised as a concern”*  *“I didn’t have the same midwife for long enough to speak to them, it was rather stressful and upsetting”*   1. preference for self-management   *“I don’t like to talk about it and think I can manage on my own”*  *“I just wanted to deal with it myself”*  *“I don’t really like to talk about it I have had some sort of disordered eating for a very long time it is very much part of me and no one else’s business”*   1. current ED symptomatology   “I didn’t think it was relevant as I have been OK for a few years now”  “It wasn’t affecting me during my pregnancy, it helped”  “I felt like I was a lot better when I fell pregnant”   1. illness awareness   *“Binge eating doesn’t seem like that big of an issue and I’ve never seen it as an eating disorder before*”  *“I have only really just recognised that I have an issue & at the time I was pregnant did not realise. I just thought I was a greedy person”* | Wish for support  Wish for support  Self-help strategies  Self-help strategies  No wish for support  No wish for support  No wish for support  No wish for support  No wish for support  No wish for support | Obstacles  Obstacles |
| Tierney et al. (2011)    Treading the tightrope between motherhood and an eating disorder: A qualitative study | Four themes:   1. fear of failure   *‘‘I really, really wanted to be able to eat more in order to produce better milk and I couldn’t do it and I felt that I’d failed him. I can remember crying when I was buying the formula for the first time and I couldn’t. . .be around when he was given the formula because it upset me. . .I didn’t want anyone else to know he was getting the formula. There was only me and my husband that knew we were giving it to him.’’*  *‘‘I’m free now aren’t I really. I’ve not got that physical attachment to [daughter]. We’re two separate entities. My responsibility to her is in a different way now, it’s not in that physical, nutritional type.’’*  *‘‘. . .he wasn’t a nightmare baby but they cry a lot don’t they the first few months. . .I don’t like it if he starts to cry in front of other people because I always think he’s a problem and he’s not, he’s a baby, but I just feel on edge and that sends me on edge...’’*  *‘‘I think people who haven’t experienced it can be very judgemental of someone who’s pregnant and can’t eat properly. I took part in an article for [newspaper] last year about eating disorders in pregnancy and although I had a lot of very positive feedback and supportive comments from my family and friends, over the internet there were a lot of really nasty comments about it posted on the website ‘these selfish women, they shouldn’t have children’, things like that. . . if you haven’t got that experience then it seems, it just seems like you are putting your appearance above your child’s health and that’s how it must seem to people I think.’’*   1. transforming body and eating   *‘‘I’m really excited but at the same time I’m really scared that I’m going to be awful. . .I don’t want to class myself as having an eating disorder but at the same time I know that I have disordered thinking and. . .I’m worried that might get in the way of being able to cope with being a mum.’’*  *‘‘. . .immediately you’ve got to be careful because if you’re told there’s a certain amount you should put on, to put on more than that, well that’s just an absolute no no. . .part of it was just wanting to prove them wrong, that you didn’t have to gain that much. . .’’*  *‘‘I do eat a lot more than before I was pregnant. . .I’d never have any breakfast, never used to really eat dinner. It was always my main meal in the evening. I never used to pick during the day. I just used to live off one meal. . .I saw the midwife about eight weeks ago now and she’d said she’d found something in my urine that had shown that my body was eating my fat as energy because I wasn’t putting any fat on.’’*   1. uncertainties about child’s shape   *‘‘. . .she’s very small and the anorexic part of me likes that. She’s not skinny, she’s just very small. . .but on the other hand because she’s small I’m constantly trying to pack food into her. I think the health visitors are aware of me having an eating disorder, sometimes I feel people are constantly checking up on me, ‘am I starving her?’ this sort of thing, which it’s the opposite.’’*   1. emotional regulation   *‘‘well I’ve had the* *baby. . .how quickly can I get back to. . .doing my exercises. . .?’’*  *‘‘I’d stopped like proper exercising, weights and biking and stuff like that at about six months and then I thought OK we’ll just see this as a little retirement. You can start it all up again once the baby’s here. And I just let go fullstop then. I just thought OK just monitor your weight, make sure you don’t put too much on and just be relaxed about it all. You can work at making it all better once [daughter] turns up.’’*  *‘‘I think it was because my stomach was getting much bigger. . .I mean obviously I was growing because of the baby but when I look back I didn’t have a huge bump. . .but at the time it seemed massive. So I was, I suppose, I wasn’t thinking of the baby, I was just seeing my stomach was fat so I was trying to get the fat out.”*  *‘‘I want to be consumed by [daughter] to a certain degree but I still want a bit of me too. . . I remember going to the gym for the first time, I was still breastfeeding her and I started crying just because I thought I’m still there. I felt like I’d lost me. It was really weird, it was surreal.’’* | Whish for support  Self-help strategies  Self-help strategies | NA  Vulnerability  Vulnerability  Vulnerability  Vulnerability  Vulnerability  Vulnerability  Vulnerability  Vulnerability |
| Claydon et al. (2018)    Waking up every day in a body that is not yours: a qualitative research inquiry into the intersection between Eating Disorders and pregnancy. | Six themes:   1. control   *“That was the hardest for me, because at the end of the day, I didn’t have those numbers to look back on. I didn’t know how I did that day. I didn’t know like any of that so, it was a complete kind of loss of control and having to give that up because otherwise it was going to completely drive me insane.”*  *“I had that false, grandiose thought that I was in control of my body and I was calling the shots. And I wasn’t and it was kind of surprising. It was a humbling experience … I can’t just march ahead and not take any precautions thinking that I can just starve my body into compliance.”*   1. disclosure to others   *“I kind of just latched onto an eating disorder, ‘cause [sic] it was my best friend, my little secret, it was just mine.”*  *“I have never sought treatment and I am incredibly private about it and sort of like my one secret that I’ve always had and it’s sort of one of those things, like they can take everything else away from me, but they can never take this away.”*  *My husband has no conception of it. He knows a little bit about that I had an eating disorder and that I've yo-yo dieted and things like that, but he really doesn't understand the mental processes that go behind it, and the constantly being, I'm going to say obsessed.*  *“I wanted no treatment, no therapy, nothing like that. My OBs are physical doctors … if I could physically take care of it myself, there was no need for me to involve them.”*  *“My doctor never knew any of this because I kept everything. Everything. No one knew anything. No one in the whole world. Only me.”*  *“I certainly felt a lack of … communication between psychiatric care and maternity care and needing some sort of, it doesn't have to be a specialized midwife but just someone who can cross barriers and help you navigate your way through the pregnancy from both perspectives and not just one or the other.”*   1. battle between mothering & eating disorder   *“I swear to god, I had like a countdown to when she was born, and I didn’t have to eat anymore.”*  *“the guilt of not being able to get myself together for the sake of the baby.”*  *“It becomes that battle where you're like well, I've got morning sickness. Should I use that as an excuse? I'm not going to say that I didn't do it once or twice, but it was mainly due to the fact that I was already throwing up anyway.”*  *“Rosie Robot was who carried the babies, but when you think about a robot carrying a baby, they don't have any emotion. There's nothing there. Then there was me who was trying to be me. I tried. I didn't know I was doing this stuff at that time. I had no idea. I never wore any maternity clothes, ever. I wouldn't wear maternity clothes. I hid it. I hid my pregnancy. Our next-door neighbors … didn't know I was pregnant until I was 9 months pregnant.”*  *“so incredibly selfish … I feel like that kind of hidden darkness could be brought out by something like pregnancy, so I don’t know how that would work.”*   1. intergenerational transmission   *“I think my hardest thing is I’m a total hypocrite, because I’m telling her you know, well as long as you are healthy, and you drink enough water and you get enough sleep, and you exercise and everything, you’re going to be fine … while at the same time, I don’t do that at all.”*  *“It’s difficult to have an eating disorder and then be in the role that I’m in because I feel you know, it’s the imposter syndrome, right like how am I trying to guide these kids into healthy decision making when secretly I’m you know, I’m participating in my own kind of battle?”*  *“I’m sort of scared of having girls … I mean like it’s a stereotype because guys can also have eating disorders. But like I’m more scared of having girls like, being a mother to girls.”*  *“I've done a lot of reading about this, I think part of the reason that I feel like I have a weight problem is because my mother had a weight problem, and I was never really able to accept myself as beautiful. Just from this body image perspective ... I never learned what was healthy.”*  *“When I started dieting in middle school, she [Louise’s mother] actually taught me how to diet. She taught me how to count calories. She taught me how to weigh food, and how to prepare food, and encouraged me in dieting until it became a little more obvious that it was out of hand. I mean obviously, she didn’t mean for that to happen.”*  *“Fortunately, my husband, when they were in second grade and third grade … because I was making like bizarre food. He completely took over food. He brought in chips. He normalized food for my girls. I think that probably saved them, considering that my own biological mother had an eating disorder and then I did.”*   1. weight and body concerns   *“objectively I know that people gain weight in pregnancy, but I think another fear would be how I would lose it afterward … like how to do it in like the healthiest way possible.”*  *“feeling like you have to lose weight is definitely a trigger for an eating disorder.”*  *“somebody could know and do the checks, but it’s information that I’d rather not know.”*  *“it didn’t feel as revealing the way I think being weighed when not pregnant does … it was kind of a relief … it didn’t really mean that I was a big, fat pig.”*  *“I feel so fleshy, swollen, flabby, jiggly, sloppy, oily, nasty, just FAT. It’s fucking killing me. I miss restricting so badly … this body is like a prison and I want out.”*  *“This is going to sound terrible, but I’m not going to be one of those girls who look pretty when they’re pregnant. They’ve got this nice slender body, and then they just have this little bump … No, mine’s going to be one of those like, “Well, I look like a marshmallow,” because that’s just how my body* *carries weight … I know that that’s how I’m going to look, and how I’m going to feel, and it scares me.”*   1. coping strategies   distraction as a coping mechanism, as well as reframing the pregnancy as a “temporary medical condition.”  *“I completely threw myself relentlessly into school at that point … because I had to distract myself somehow.”*  *“I forced myself to not keep track of things anymore … because at the end of day I knew I had to be consuming at least 2300 calories and if I hit that, I’d be upset, but then if I didn’t hit that, I’d be upset. So, either way, you’re never going to win in that situation … I just had to give up accountability all together and just say fuck it.”*  *“I don’t trust myself to make the best decisions regarding pregnancy. Maybe I would and I’m underestimating myself, but I would rather just have the outside support.”*  *“I go to the bathroom at the end of the meal and it's like I have to remind myself not to do it. I choose not to because I know it's not healthy, but it's a choice that I make.”* | No wish for support  No wish for support  No wish for support  No wish for support  Wish for support  No wish for support    Support from a partner  Self-help strategies  Self-help strategies  Wish for support  Self-help strategies | Vulnerability  Vulnerability  Vulnerability  Vulnerability  Vulnerability  Vulnerability  Vulnerability  Vulnerability  Vulnerability  Vulnerability  Vulnerability  Vulnerability  Vulnerability  Vulnerability  Vulnerability  Vulnerability  Vulnerability  Vulnerability  Vulnerability |
| Mason et al.  (2012)  The experience of pregnancy in women with a history of anorexia nervosa: An Interpretive Phenomenological Analysis | Four themes (***subordinate themes***):   1. effortful resistance of AN   ***Persistence of AN psychopathology :****“It’s there, and it’s always going to be there, you know, I think once you’ve had an eating disorder, you always have an eating disorder, I don’t think there’s any cure, because it’s a... a mental illness rather than anything else, isn’t it?”*  *“Um... the only thing that I did manage to do, mostly, was give up taking laxatives.”*  *“I just tried to ignore them. They were still there, I was watching the scales go up, and it was horrible... Yeah... It’s just horrible watching them go up, and up, and up. I felt very out of control of it.”*  *“...as I began to lose weight, after I had him, the fatter I started feeling. And as I lost more and more weight, I guess I just... felt huge. And I went and got all these diet books, and I was like I‘m going to lose the weight, I was so determined to lose the weight.”*  ***Motivation for change:*** *“Yeah, I did struggle with the changing body, yeah, I did find that hard… , but at the end of the day I knew that was what I wanted. To have kids. So I had to motivate myself to do it.”*  *“...You’re not really thinking about anyone else, and all you think about is food, and so... um... I just had to stop being like that, it was like ‘‘well I can’t think about myself anymore, I’ve got a baby that I need to, that needs to develop and it needs to be born and it needs to be perfect, and so I can’t think about myself anymore.”*  *“And it wasn’t just my baby, it was husband’s baby as well, and I didn’t want to let anyone down...”*  *“Just being scared I’d kill her. By not eating enough. And then it would all be my fault.”*  ***Strategies used “for the duration”:*** *“I always struggle with the inner voice more towards the evening times. So during the day I suppose I was just so busy anyway, it was a lot easier to eat, so I tended to have more of my food during the day and then I didn’t have to worry in the evening.”*  *“I didn’t react to [the feelings], that’s the important thing. I didn’t let them fester too much. I think the feelings were there, but I didn’t respond to them…”*  *“And the sort of eating thing, and how I eat, that could just sort of be kept on the backburner until after I’d had the baby. And then I’d start thinking about it again and how I was going to get, you know, lose the weight and get back to how I was before.”*  *“I think I almost... every time a feeling crept in, I almost saw it as a bag that I put to one side for the time being. It was like, you know, that’s an issue for later, that’s going to have to be dealt with later...”*  *“I was fighting the ED more, I was more prepared to challenge it, and I was more worried about the consequences”*   1. The unvalued self, valued other dialectic   ***Acceptability of AN for self, but not for baby:*** *“I suppose it I do it now as a way of, well, just punishing myself and things like that, but I suppose when you’re pregnant you don’t want to punish the child as well.”*  ***Acceptance of ‘pregnancy’, rejection of ‘fat’:*** *“I was OK with the changes which I could directly attribute to being pregnant. I was fine with having a big bump, and I quite liked that. Um... but I wasn’t OK with putting weight on anywhere else at all... ...I was happy to look pregnant, and I wanted to look pregnant, I just didn’t want to have any fat anywhere else, on my legs, or arms, or face, anything like that...”*   1. In new territory   ***Unfamiliar lack of control:*** *“It’s kind of like… you’re gaining weight, but you’re totally powerless to do anything about it. You just gain loads of weight, well I do, when I’m pregnant, without doing anything to gain weight... I think your body’s just not in control at all when you’re pregnant. It just does what it likes, and you can’t do anything about it.”*  *“I think when things feel out of control you just do what you can to get it back, and that might mean being more, more rigid and restricting more, and I did get very – when I was pregnant with Josh – I did get very set in a routine.”*  ***A new meaning of embodiment:*** *“I just knew that that was going to be the precious, the most precious thing to look after, and actually my body was sup-* *porting and nurturing a little person in there, and that was more important to me than anything.”*  *“It was like it was her body, not my body”*  *“I didn’t feel like a pregnant woman because... I had a couple of friends who were pregnant at the same time, and, um... it was so different.. I felt like I wasn’t, I wasn’t a proper mum, or I wasn’t a proper expectant mum.... ...I couldn’t really feel pregnant... ...I liked the fact that I could exercise, and I felt, um... I think it made me feel quite strong, and like I could fight my body, and I could overcome this, I don’t have to lie down because I’m pregnant. But at the same time I wanted to feel pregnant. Um... and I wanted to be able to do what everyone else was doing.”*   1. Feeling distanced   ***Unmet emotional needs:*** *“So I didn’t get any help. And I did actually say to my doctor as well, I remember saying to him I felt really depressed and low, and I wasn’t offered any help... I felt as if, ‘do people really believe me here? Do people believe that I feel...?’... And so I felt, yeah, I felt wretched... Yeah I didn’t feel good. I felt completely and totally miserable when I was pregnant.”*  ***Isolation:*** *“I guess I felt as if... I had been abandoned to be honest... I wasn’t asking for a lot, I think. And that’s the thing that I feel so let down about. I wasn’t asking for a lot, I was just asking for a phone call, or someone to just pop by and see me, and just sit and chat for an hour, you know. I just wanted some contact with the outside world, and I’m not exaggerating when I say that just didn’t happen at all.”*  ***Professional support:*** *“...a lot of what the midwives give you is geared towards staying active and not gaining too much weight during pregnancy, and all the health problems which could be caused by gaining too much weight. It’s aimed at the general population, and I can see that now. But... I think when you’re in the middle of an eating disorder, you could sort of use it to think ‘‘well, it’s just as unhealthy if I gain all this weight, and if I gain weight I’ll have gestational diabetes and pre-eclampsia and all these things”.”* | Self-help strategies  Self-help strategies  Self-help strategies  Self-help strategies  Self-help strategies  Self-help strategies  Self-help strategies  Self-help strategies  Self-help strategies  Self-help strategies  Self-help strategies  Self-help strategies  Wish for support  Wish for support  Wish for support | Vulnerability  Vulnerability  Vulnerability  Vulnerability  Vulnerability  Vulnerability  Vulnerability  Vulnerability |
| Stitt & Reupert (2014)  Mothers with an eating disorder: “food comes before anything” | Six themes (***subordinate themes***):   1. impact of the parents’ eating disorder on children   ***social isolation:*** *“I would avoid things where there was food”*  *“I just can’t be in that situation. . . . so I deny my kids going there”*  *“I can always find an excuse why we can’t go somewhere”*  *“I ended up putting my daughter in day care for the simple reason that she needed socialization just one day a week because I wasn’t able to take her to playgroup and those sorts of activities.”*  *“. . . . my illness has had a massive financial blow to our family and that’s affected what they can do socially”*  *‘****My head’s too full’: Emotional absence from their children:*** *“.... because my head’s too full. . . . from [what] I’ve had to eat and my head’s too full with all the negative aspects of that . . . .”*  *“. . . . it would be nice to be able to be just 100% into enjoying my children . . . [instead of] all I’m ever thinking about is, ‘Oh my God, oh my God. What have I eaten? When am I going to eat next? What am I going to do?’ – always thinking about that.”*  *“. . . . how much time you spend in your head obsessing about your weight or how you look, or whether you’ve eaten, or what you’ve eaten, if you look back you probably lost time that you could of just spent doing things with the kids . . . . I sort of wonder, how much times did I lose, or, how many times did I snap at them, how many times was I unfair?”*  ***‘It’s easier to say yes’: Problems setting boundaries:*** *“. . . . I was so caught up in my ED that I just let him get away with stuff and just not really do anything ....I was more interested in keeping him quiet, so I could go and exercise .... burn calories.”*  *“Great – I don’t have to eat tonight .... there is no obligation [to eat].”*  *“. . . . I don’t know what’s the best thing for me to say because I don’t know if it’s coming from my ED voice or if it’s actually coming from a normal mum.”*  ***The parentification of children:*** *“. . . . not so much my parenting but them parenting me and worrying about me”*  *“. . . . C’mon, up you get, let’s go and have a shower . . . .”*  *“. . . . C’mon mum you have to get up to get the boys ready.”*  ***The impact of younger versus older children:*** *“. . . . I’m lucky that they are young, because it doesn’t impact [on] them quite so much.”*  *“.... when they’re little it’s a lot easier to get away with it . . . .”*  *“. . . . it’s impacted my children in their later adolescence and early adulthood more than it probably did through their developing years.”*   1. modelling disturbed eating behaviours   *“Probably the biggest thing is that what if they copy .... it’s my coping thing and I’m scared that they might see .... okay, well Mum does this to cope so we will too.”*  *“. . . . not wanting any of my bad behaviour to rub off on her [child] and influence her in any way whatsoever”*  *“. . . . in denial about my own condition .... enough awareness there that I wanted to be very careful not to, I guess, pass my demons on”*   1. food comes before anything   *“. . . . I don’t want my children to worry but the disorder overrides all of that”*  *“.... because in my ED mind, the food comes before anything – the love of the kids, anything. It’s all about the food and losing weight”*  *“.... like I have my routine that yeah, and I get angry [when her children interrupt her] .... when it’s time to purge and it’s like what am I going to do now? Yeah I get really frustrated and try and get them out as soon as I can.”*  *“. . . . it [binging and purging] was getting quite regular and they were aware of it and I wasn’t comfortable with them knowing mummy’s always sicky sick [expression used by the family to explain the purging] . . . . I had to change it and in my weird twisted mind, the way I changed it was by stopping eating, which is not so much of an issue in terms of family life ....”*   1. ‘There would be no world without them’: children motivate recovery   *“I don’t want my kids growing up with a mother who’s dysfunctional in any way . . . . I’m dealing with it for their sake . . . . the inspiration and drive for me to fix myself is so that they can grow up in a healthy environment. If it hadn’t been for him, I would probably be, either dead or still well and truly in relapse, to be honest.”*  *“.... around them [children] leaving home and not having the focus on you know, going to work, coming home, cooking dinner.”*  *“They really keep me normal.”*   1. secrecy   *“I would do it [purge] even if they could hear me .... obviously at age four and five they don’t understand what I am doing . . .”*  *“I don’t think they know .... they haven’t mentioned anything”*  *“. . . . I haven’t seen any signs that he knows . . . .”*  *“I’m also diabetic so anytime things might come up to do with the medical side of things, they think it’s because of Mum’s diabetes .... it’s like this massive lie I live .... I do it ultimately to protect my kids, they couldn’t handle knowing ....”*  *“[after visiting participant in hospital] . . . . he became quite concerned about my eating, and [husband] and I had a talk about that [ED] and about making that something that’s not discussed in front of the kids .... He [son] was really stressed about it and that’s not okay for a 10 year old to have that sort of burden.”*  *“.... when she got old enough [five years of age] to realise what was going on and why I was the way I was, we [participant and husband] talked to her and explained everything so she had a good understanding of things.”*  *“[did] not want any of my bad behaviour to rub off on her and influence her”*   1. treatment needs   *“.... directed at younger people and not older people . . . yeah there isn’t [aren’t] methods that include family life”*  *“meal plans within the context of family life”*  *“I can’t set my meal plans .... when you’ve got kids your life isn’t your own to manage as you would like ....”*  *“It’s not appropriate for me as an adult to have my mother come and take control of my food, and while my husband, I guess, was willing and able to do that, that’s sort of not really an appropriate balance in the relationship either.”*  *“.... therapists don’t quite get that everybody doesn’t have babysitters they can ring up at short notice .... I actually had one woman who .... basically said ‘If you can’t commit to come every single week then I can’t treat you’ and I said ‘Look, I’m trying but if my kids are sick, what do I do’ ....”* | Wish for support    Wish for support  Support from a partner  Wish for support | NA  NA  NA  NA  NA  NA  NA  NA  NA  NA  NA  NA  NA  NA  NA  NA  NA  Obstacles |

^a^ Quotes that wasn´t about wanted support but still relevant for women with ED during pregnancy were classified as Obstacles or Vulnerability. Quotes not relevant at all were code not applicable (NA
